# Resilience, condom use self-efficacy, internalized homophobia, and condomless anal sex among black men who have sex with men, New York City

**DOI:** 10.1371/journal.pone.0215455

**Published:** 2019-04-24

**Authors:** Erica L. Dawson, Maria C. B. Mendoza, Zaneta Gaul, William L. Jeffries IV, Madeline Y. Sutton, Patrick A. Wilson

**Affiliations:** 1 Division of HIV/AIDS Prevention, National Center for HIV/AIDS, Viral Hepatitis, STD, and TB Prevention, Centers for Disease Control and Prevention, Atlanta, Georgia, United States of America; 2 Epidemic Intelligence Service, Centers for Disease Control and Prevention, Atlanta, Georgia, United States of America; 3 ICF, Atlanta, Georgia, United States of America; 4 Mailman School of Public Health, Columbia University, New York City, New York, United States of America; New York Blood Center, UNITED STATES

## Abstract

**Background:**

Black men who have sex with men, who account for less than 1% of the U.S. population, account for approximately 25% of new HIV infections annually. Condomless anal sex contributes to HIV infection among black men who have sex with men. The capacity to recover quickly from difficulties (resilience) may be protective against condomless anal sex, but has been understudied among black men who have sex with men. Psychosocial factors related to resilience, i.e., condom use self-efficacy and internalized homophobia, may also affect condomless anal sex. We assessed the association between resilience, condom use self-efficacy, internalized homophobia and condomless anal sex among black men who have sex with men.

**Methods:**

Data are from a 2010–2011 study examining condomless anal sex (past 60 days) among black men who have sex with men in New York City. Validated scales assessed resilience (theoretical range = 0–100), condom use self-efficacy (theoretical range = 27–135), and internalized homophobia (theoretical range = 9–36). We described continuous variables using median and interquartile range (IQR). Univariable and multivariable Poisson regression models assuming a robust variance estimator were used to compute unadjusted and adjusted prevalence ratios, respectively, and their corresponding 95% confidence intervals (CI). Adjusted prevalence ratios (aPR) examined the association of resilience, condom use self-efficacy, and internalized homophobia with condomless anal sex, while controlling for potential confounders (e.g., having >1 sex partner).

**Results:**

The median resilience score within our sample (N = 228) was 75 (IQR = 66–83). Many black men who have sex with men reported condomless anal sex (55.7%) and >1 sex partner (58.8%). Decreased condomless anal sex was associated with increased levels of condom use self-efficacy (aPR: 0.94 per 10-point increase in condom use self-efficacy score; CI: 0.90–0.97; *p*-value: 0.001). Condomless anal sex was not associated with resilience or internalized homophobia.

**Conclusions:**

Within this sample of black men who have sex with men, condomless anal sex was prevalent. Greater resilience was not protective against condomless anal sex. Interventions that support condom use are warranted for black men who have sex with men.

## Introduction

In 2016, gay, bisexual, and other men who have sex with men (MSM) accounted for 66.8% of all new HIV diagnoses in the United States (U.S.) [1]; black/African American MSM (BMSM) are disproportionately affected by HIV infection, accounting for 38.5% of MSM new diagnoses in 2016 [1]. The New York City (NYC) HIV epidemic is similar to what we see nationally; MSM accounted for 54.2% of all new HIV diagnoses in 2016, and BMSM represented 18.0% of new HIV diagnoses in NYC compared with 11.1% for white MSM [2].

Condomless anal sex is the primary risk factor for sexual HIV transmission among MSM [3, 4]. However, BMSM are more likely to report condom use during sexual activity and less likely to report some HIV risk factors, (i.e., high numbers of male sex partners or use of drugs associated with HIV infection), compared with their non-black MSM peers [5]. Data also show that BMSM are disproportionately affected by structural and psychosocial barriers, such as unemployment, low income, history of incarceration, and depression [5, 6]. These data underscore that structural and psychosocial factors also play a role in HIV infection among BMSM, and warrant examination with condomless anal sex as factors that contribute to increased risk for HIV infection among BMSM.

One psychosocial factor that may be protective against condomless anal sex is resilience. Resilience is the ability to recover quickly from stressful life situations; it is associated with psychological well-being and can potentially reduce HIV risk behaviors by buffering the effects of stigma and social stressors that contribute to condomless anal sex among MSM [7–9]. Data from mostly young black, Hispanic/Latino, and Native American MSM suggest that social supports, including family and house-ball communities (informal community networks of lesbian, gay, bisexual, transgender, and queer people), serve as sources of resilience [10–11]. A study with older, HIV-infected MSM showed that resilience was positively associated with improved health-related quality of life in later years [12]. Data also suggest that resilience may be protective against some syndemic factors that promote HIV infection among BMSM (e.g., depression, substance abuse, and childhood sexual abuse) [13,14].

Though studying resilience has been recommended by the National Institute of Health’s Lesbian, Gay, Bisexual, and Transgender Research Coordinating Committee (2013) to find out “how it develops, may protect health, and may buffer against the internalization of stigma and/or other negative experiences associated with sexual or gender minority status” [15], few studies have fully examined resilience and its relationship to condomless anal sex among BMSM. Data from two U.S. studies with BMSM suggest higher resilience may be associated with decreased condomless anal sex [13, 16]. However, other studies indicate no association between resilience and condomless anal sex. Additional data are warranted to inform the question of association between condomless anal sex and resilience among BSMSM and the development of future resilience-focused HIV prevention interventions.

One challenge with studying resilience in relation to condomless anal sex is that it is associated with other, interrelated psychosocial factors that affect BMSM’s risk for HIV infection, such as condom use self-efficacy and internalized homophobia. For example, lacking condom use self-efficacy can promote condomless anal sex by making BMSM feel doubtful about their abilities to use and discuss condoms with sexual partners [17]. Moreover, internalized homophobia, which is associated with decreased resilience levels [18], may promote condomless anal sex by diminishing BMSM’s perceived self-worth [19]. Internalized homophobia could also confound the association between resilience and condomless anal sex. However, resilience in the form of resolution of internalized homophobia has been associated with positive health outcomes [20]. Internalized homophobia is an important covariate to consider in light of it being associated, albeit weakly, with condomless anal sex among some MSM [21]. We hypothesize that condomless anal sex is associated with resilience, in the presence of condom use self-efficacy and internalized homophobia.

To better understand resilience and inform HIV prevention research gaps, we examined resilience, condom use self-efficacy, internalized homophobia, and condomless anal sex within a sample of BMSM in NYC. Our goal is to provide data that may be useful in development of strengths-based HIV prevention interventions for BMSM.

## Methods

### Recruitment

The Brothers Connect Study was a cross-sectional study that examined the condomless anal sex and other health-related behaviors of BMSM in NYC during 2010–2011. Details have been published previously [22]. Briefly, a sample of 228 young BMSM were recruited from social spaces, websites, and community based organizations, using a convenience sample, and enrolled in the study. Eligibility criteria included a self-report of African American or black race, male sex at birth, 18–30 years of age, residence in the NYC or northern New Jersey area, and oral or anal sex with a man in the past 60 days. Participants completed a self-administered web-based survey, which contained questions on demographic characteristics, sexual behaviors, psychological constructs (e.g., resilience, condom use self-efficacy, and internalized homophobia), and lifetime HIV testing. The study protocol was approved by the Columbia University Institutional Review Board and the Centers for Disease Control and Prevention’s project determination process. All participants provided signed informed consent.

### Measures

#### Condomless anal sex

Our dependent variable was condomless anal sex, which was defined as having receptive or insertive anal sex with a man without a condom in the past 60 days.

#### Resilience

We used the 25-item Connor-Davidson Resilience Scale [23]. Sample items included “You are able to adapt to change”; and “You tend to bounce back after illness or hardship”. Response options ranged from 0 (not true at all) to 4 (true nearly all of the time). Scale ratings were based on how the subject felt over the past month. The responses from the 25 items on the scale were summed to create the total resilience score. Total resilience scores range from 0–100, with higher scores indicating greater resilience. Previous studies have shown the Connor-Davidson Resilience Scale to be reliable with BMSM in NYC and Jackson, Mississippi [9, 16]. The scale had high internal reliability within this sample (Cronbach’s α = 0.97).

#### Condom use self-efficacy

We used the 27-item Condom Use Self-Efficacy Scale, which measures one’s confidence with using a condom or asking sexual partner(s) to wear a condom [24]. Sample items include “I can say no to sex with a new partner if we don’t have a condom even if I want to have a relationship” and “I can say no to sex if my partner and I don’t have a condom even if we have not used one in the past.” Response options ranged from 1 (not sure) to 5 (completely sure). The responses from the 27 items on the scale are summed to create the total condom use self-efficacy score. Assuming that the total condom use self-efficacy score includes a response from each item on the scale, scores range from 27–135. Higher scores indicate higher condom use self-efficacy. The scale had high internal reliability within this sample (Cronbach’s α = 0.97).

#### Internalized homophobia

The Internalized Homophobia Scale (Ego-Dystonic Homosexuality Scale) is a 9-item scale designed to measure the extent to which lesbian, gay, and bisexual men and women do not accept their sexual orientation, are uneasy about their same-sex desires, and seek to avoid encounters with other sexual minorities [25]. Participants were presented with statements such as “I often feel it best to avoid personal or social involvement with other gay/bisexual men,” “I wish I weren’t gay/bisexual,” and “I feel that being gay/bisexual is a personal shortcoming for me” and were asked to rate how true these statements are for them. Response options ranged from 1 (never) to 4 (often). The responses from the 9 items on the scale are summed to create the total internalized homophobia score. Assuming that the total internalized homophobia score includes a response from each item on the scale, scores range from 9–36, with higher scores indicating greater internalized homophobia. The scale had high internal reliability within this sample (Cronbach’s α = 0.88).

### Covariates

Selected covariates included the following demographic factors: age, race (African American/black, black Hispanic/Latino, mixed race, and Afro Caribbean/West Indian), educational attainment (≤ high school, > high school), annual household income (≤ $10,000, > $10,000), current employment status (unemployed/disability or other, working or student), and relationship status (single, married or have a boyfriend/girlfriend). Health-related covariates included health insurance status (insured, uninsured), number of sex partners in the past 60 days (1 partner, >1 partner), lifetime HIV testing (yes, no), self-reported HIV status (negative, positive, don’t know), and current use of psychotropic medication (yes, no). We also measured sexual identity (gay/homosexual, bisexual/heterosexual/straight/other), and lifetime incarceration history (yes, no).

### Statistical analysis

To compare the prevalence of condomless anal sex among BMSM, we used univariable and multivariable Poisson regression models with a robust variance estimator, and obtained unadjusted and adjusted prevalence ratios (aPR) and their corresponding 95% confidence intervals (CI). Unadjusted prevalence ratios (PR) measured the individual association between condomless anal sex and each of the characteristics. Characteristics with a *p*-value <0.10 in the unadjusted regression analyses were considered as candidate variables in the multivariable model. Backward selection was used to determine the final adjusted model, which investigated the association of internalized homophobia, condom use self-efficacy, and resiliency with condomless anal sex, adjusting for all remaining factors that were statistically significant at the 0.05 level. Note, unadjusted and adjusted prevalence ratios and 95% CIs for internalized homophobia, condom use self-efficacy, and resiliency reflect a 10-point increase in each scale score. Statistical analysis was performed using SAS Version 9.4.

## Results

Data for 228 BMSM were available for this analysis. Table 1 summarizes the characteristics of study participants. The median age of participants was 24.1 years; 62.7% had acquired more than high school education; 39.3% were unemployed or disabled; and 79.0% had health insurance. Additionally, 74.1% self-identified as gay or homosexual; 98.3% had been tested for HIV at least once during their lifetimes; and 24.1% had ever been incarcerated. Of 228 participants, 55.7% reported having condomless anal sex in the past 60 days, 58.8% reported having >1 sex partner, and 23.7% reported being HIV positive. Among participants reporting >1 sex partner in the past 60 days, 64.2% reported having condomless anal sex. The median resilience score was 75 (IQR: 66–83), the median condom use self-efficacy score was 115 (IQR: 96–128.5), and the median internalized homophobia score was 30 (IQR: 25–34).

**Table 1:** Characteristics of study participants, brothers connect study, New York City,2010–2011.

Characteristic	n (% of Total^a^)	Number Reporting Condomless Anal Sex, (% of n)
**Age, median (IQR)**	24.1 (21.2–28.3)	24. 6 (21.4–28.1)
**Race/ethnicity**		
African American/Black	137 (61.7)	69 (50.4)
Hispanic/Latino Black	43 (19.4)	28 (65.1)
Mixed race	28 (12.6)	17 (60.7)
Afro Caribbean/West Indian	14 (6.3)	8 (57.1)
**Educational attainment**		
High school or less	85 (37.3)	48 (56.5)
Greater than high school	143 (62.7)	79 (55.2)
**Income**		
≤ $10,000	121 (53.1)	73 (60.3)
>$10,000	107 (46.9)	54 (50.5)
**Employment**		
Unemployed/disability or other	84 (39.3)	53(63.1)
Working or student	130 (60.8)	66 (50.8)
**Health insurance**		
Insured	180 (79.0)	104 (57.8)
Uninsured	48 (21.1)	23 (47.9)
**Relationship status**		
Single	167 (73.3)	94 (56.3)
Married or have boyfriend/girlfriend	61 (26.8)	33 (54.1)
**Number of sex partners**		
1 partner	94 (41.2)	41 (43.6)
>1 partner	134 (58.8)	86 (64.2)
**Sexual identity**		
Gay/homosexual	166 (74.1)	91 (54.8)
Bisexual/heterosexual/straight	58 (25.9)	34 (58.6)
**Ever tested for HIV**		
Yes	224 (98.3)	126 (56.3)
No	4 (1.8)	1 (25.0)
**Self-reported HIV status**		
Negative	169 (74.1)	92 (54.4)
Positive	54 (23.7)	32 (59.3)
Unknown	5 (2.2)	3 (60.0)
**Psychotrophic medication**		
Yes	24 (10.6)	16 (66.7)
No	203 (89.4)	110 (54.2)
**Ever incarcerated**		
Yes	55 (24.1)	35 (63.6)
No	173 (75.9)	92 (53.2)
**Resilience, median (IQR)**	75 (66–83)	73 (66–82)
**Condom use self-efficacy, median (IQR)**	115 (96–128.5)	111 (90–126)
**Internalized Homophobia, median (IQR)**	30 (25–34)	30 (25–33)
**Condomless anal sex (yes)**	127 (55.7)	—

IQR, interquartile range

^**a**^Categories may not add up to N = 228 due to missing data

Condomless anal sex prevalence for characteristic subgroups are in Table 1 and their corresponding unadjusted prevalence ratios in Table 2. Condomless anal sex was associated with condom use self-efficacy (PR: 0.93 per 10-point increase in condom use self-efficacy score; CI: 0.90–0.96; *p*-value: <0.001). Neither resilience nor internalized homophobia were associated with condomless anal sex. Employment and number of sex partners had *p*-values <0.10 in univariable regression analyses, and were therefore considered as candidate variables for entry into the multivariable model.

**Table 2:** Poisson regression analysis results of HIV risk factors associated with condomless anal sex, Brothers Connect Study, New York City, 2010–2011.

Characteristic	Unadjusted Prevalence Ratio (95% CI; *p*-value)	Adjusted Prevalence Ratio^a^ (95% CI; *p*-value)
**Age**	1.00 [0.98–1.04; 0.572]	
**Race/Ethnicity**		
African American/Black	Referent	—
Hispanic/Latino Black	1.29 [0.98–1.70; 0.067]	—
Mixed race	1.20 [0.86–1.70; 0.283]	—
Afro Caribbean/West Indian	1.13 [0.70–1.84; 0.609]	—
**Educational attainment**		
High school or less	Referent	—
Greater than high school	0.98 [0.77–1.24; 0.857]	—
**Income**		
≤ $10,000	1.20 [0.94–1.52; 0.140]	—
>$10,000	Referent	—
**Employment**		
Unemployed/disability or other	1.24 [0.98–1.57; 0.070] ^b^	—
Working or student	Referent	—
**Health insurance**		
Insured	Referent	—
Uninsured	0.83 [0.60–1.14; 0.252]	—
**Relationship status**		
Single	Referent	—
Married or have boyfriend/girlfriend	0.96 [0.74–1.26; 0.771]	—
**Number of sex partners**		
1 partner	Referent	Referent
>1 partner	1.47 [1.13–1.91; 0.004]^b^	1.33 [1.01–1.76; 0.044]
**Sexual identity**		
Gay/homosexual	Referent	—
Bisexual/heterosexual/straight	1.07 [0.83–1.38; 0.609]	—
**Ever tested for HIV**		
Yes	Referent	—
No	0.44 [0.08–2.44; 0.350]	—
**Self-reported HIV status**		
Negative	Referent	—
Positive	1.09 [0.84–1.41; 0.523]	—
Unknown	1.10 [0.53–2.28; 0.794]	—
**Psychotropic medication**		
Yes	Referent	—
No	0.81 [0.60–1.11; 0.190]	—
**Ever incarcerated**		
Yes	1.20 [0.94–1.53; 0.149]	—
No	Referent	—
**Resilience**^c^	0.94 [0.86–1.03; 0.192]	1.02 [0.92–1.12; 0.754]
**Condom use self–efficacy**^c^	0.93 [0.90–0.96; <0.001]	0.94 [0.90–0.97; 0.001]
**Internalized homophobia**^c^	0.89 [0.75–1.07; 0.211]	0.95 [0.80–1.14; 0.614]

CI, confidence interval

^a^Model determined using a backward selection includes number of sex partners, resilience, internalized homophobia, and condom use self-efficacy; em dashes indicate that the characteristic was either not considered for entry into the final multivariable model or after entry, was found to not be statistically significant, and therefore not included in the final multivariable model

^b^Indicates characteristics with *p*-value<0.10 in the univariable model

^c^Prevalence ratios and confidence intervals for resilience, internalized homophobia, and condom use-self efficacy are per 10-point increase in each scale score

Results of the final adjusted model are presented in Table 2. Having >1 sex partner in the past 60 days was associated with having condomless anal sex (aPR: 1.33; CI: 1.01–1.76; *p*-value: 0.044). Also, the prevalence of condomless anal sex decreased as condom use self-efficacy increased (aPR: 0.94 per 10-point increase in condom use self-efficacy score; CI: 0.90–0.97; *p*-value: 0.001). Neither resilience (aPR: 1.02; CI: 0.92–1.12; *p*-value: 0.754) nor internalized homophobia (aPR: 0.95; CI: 0.80–1.14; p-value: 0.614) were found to be associated with condomless anal sex.

## Discussion

Among our sample of BMSM in NYC, who had median resilience scores of 75 out of 100, resilience was not associated with condomless anal sex. This was unexpected given the known benefits that resilience has for sexual health behaviors among MSM, including BMSM [10, 11]. Though we also examined internalized homophobia and condom-use self-efficacy as parallel psychosocial factors, adding other variables and a larger sample size may be warranted in future studies to further examine this question. Other psychosocial factors influence condomless anal sex and should be considered, including discrimination for being gay [26] and using sex as a way to cope with social vulnerabilities and stressors [27]. Research with BMSM suggests that these factors have the potential to outweigh resilience and other protective factors [28]. Interventions addressing social vulnerabilities and stressors that place BMSM at increased risk for HIV infection could be developed in the future based on these study findings.

Condomless anal sex was prevalent among BMSM in our study. This finding is consistent with other reports of prevalent condomless anal sex among MSM [29, 30], including one study that suggests an increasing trend in condomless anal sex among MSM, including BMSM, in recent years [31]. This condomless anal sex trend may be associated with treatment optimism (defined as favorable perceptions regarding the availability or use of antiretroviral therapy in the era of treatment as prevention [TasP]), especially among serodiscordant or unknown status male partners of HIV-positive men; further study is needed.

While this cross-sectional analysis is not able to provide any data regarding possible incident HIV infections among study participants with condomless anal sex and multiple sex partners, data from past studies show that multiple sex partners and condomless anal sex are risk factors for HIV acquisition [32]. In the largest prospective cohort study of U.S. BMSM, men who reported having two or more male partners had 2.6 times the risk of HIV infection as men who reported having zero partners or one partner [4]. These data point to the need for evaluating interventions that aim to reduce the number of sex partners and condomless anal sex among BMSM even in an era in which TasP and pre-exposure prophylaxis (PrEP) are highly effective HIV prevention options. Moreover, BMSM are at disproportionately high risk for acquisition of other sexually transmitted infections that can be averted via condom use [5]. Our data suggest that continued messages about condom use are warranted.

Our study showed that the prevalence of condomless anal sex among BMSM decreases as condom use self-efficacy increases (aPR: 0.94 per 10-point increase in condom use self-efficacy score; CI: 0.90–0.97; *p*-value: 0.001), i.e., the prevalence of condomless anal sex multiplied by 0.94 for each 10-point increase in condom use self-efficacy score. This is consistent with a previous report of condom use self-efficacy being associated with increased condom use and fewer condomless sex acts among MSM [33]. Condom use may be influenced by coercive power, peer norms [34], and fear of rejection [35], especially among BMSM [36]. Data remain limited for BMSM, and future studies should further examine the construct of condom use self-efficacy for disproportionately affected BMSM.

Our study has several limitations. First, this is a cross-sectional study; causation cannot be inferred from this analysis. Second, the use of a convenience sample might have presented selection bias. Third, our sample size was small; future studies should attempt to enroll a larger number of men to strengthen analytic findings. Fourth, although we used a self-administered web-based survey to collect sensitive data, findings may be influenced by social desirability bias. Finally, our study’s use of a single-city sample limits the generalizability of our findings. This, with the narrow range of resilience variance among BMSM in our sample, may underlie our finding of a lack of association between resilience and condomless anal sex [37].

## Conclusion

In this sample of BMSM in NYC, condomless anal sex was common and was associated with having more than one sex partner and lower condom use self-efficacy. A comprehensive Cochrane review found that behavioral interventions are effective for reducing self-reported condomless anal sex among MSM, but more interventions are needed for MSM of color [38]. Efforts to reduce condomless anal sex among MSM must also be considered in the context of current public health HIV prevention messages (i.e., PrEP and promoting antiretroviral therapy to achieve viral suppression among those living with HIV infection) to be most effective. Our data suggest that developing behavioral interventions to help reduce condomless anal sex among BMSM in NYC is still warranted, and a necessary part of HIV prevention strategies.
